# Berberine inhibits angiogenesis in glioblastoma xenografts by targeting the VEGFR2/ERK pathway

**DOI:** 10.1080/13880209.2018.1548627

**Published:** 2019-01-02

**Authors:** Fa Jin, Tao Xie, Xiaoguang Huang, Xinde Zhao

**Affiliations:** Department of Neurosurgery, Zhujiang Hospital, The National Key Clinical Specialty, The Engineering Technology Research Center of Education Ministry of China, Guangdong Provincial Key Laboratory on Brain Function Repair and Regeneration, Southern Medical University, Guangzhou, China

**Keywords:** Ectopic, orthotopic, haemoglobin, CD31, Transwell, Matrigel

## Abstract

**Context:** Berberine is used in traditional Chinese medicine for thousands of years with recent reports of its anticancer activity.

**Objective:** To test antiangiogenic effects of berberine on human glioblastoma and clarify involvement of the VEGFR2/ERK pathway.

**Materials and methods:** Cell viability, proliferation and migration assays were performed to determine *in vitro* antiangiogenic effects of berberine (6.25–200 μmol/L, 6–48 h). Ectopic and orthotopic xenograft models in BALB/c nude mice were induced to determine antitumour and antiangiogenic effects of berberine (50 mg/kg by oral gavage for 28 days) or vehicle control (carboxymethylcellulose sodium).

**Results:** Berberine inhibited cell viability (IC_50_ of 42 and 32 μmol/L, respectively) and proliferation of U87 and U251 human glioblastoma cell lines. Berberine (50 μmol/L) inhibited cell migration of HUVEC by 67.50 ± 8.14% in the Transwell assay and tube formation of HUVEC by 73.00 ± 11.12% in the Matrigel assay. In the ectopic xenograft model, tumour weight was significantly decreased by 50 mg/kg of berberine (401.2 ± 71.5 mg vs. 860.7 ± 117.1 mg in vehicle group, *p* ˂ 0.001). Berberine significantly decreased haemoglobin content (28.81 ± 3.64 μg/mg vs. 40.84 ± 5.15 μg/mg in vehicle group, *p* ˂ 0.001) and CD31 mRNA expression in tumour tissue. In the orthotopic xenograft model, berberine (50 mg/kg) significantly improved the survival rate of mice (*p* = 0.0078). Berberine inhibited (*p* ˂ 0.001) the phosphorylation of VEGFR2 and ERK.

**Discussion and conclusions:** Berberine inhibited angiogenesis in glioblastoma xenografts by targeting the VEGFR2/ERK pathway. Our work sheds new light on complementary and alternative therapy for glioblastoma.

## Introduction

Glioblastoma, the most common primary brain tumour in adults with an annual incidence of 5.26 per 100,000 population or 17,000 new diagnoses per year, is usually associated with a gloomy prognosis and poor quality of life (Omuro and Deangelis 2013). The current standard of care for newly diagnosed glioblastoma is surgical resection to the extent feasible, followed by adjuvant radiotherapy (Stupp et al. 2005). Radiotherapy combined with a DNA alkylating agent such as temozolomide is regarded as the first-line adjuvant treatment for this disease (Stupp et al. 2014). However, it causes serious adverse effects such as lymphopenia and nausea, and almost all patients with glioblastoma experience disease progression (Stupp et al. 2014).

Angiogenesis is a physiological or pathological process where new blood vessels form and grow from preexisting vessels (Carmeliet and Jain 2000). Angiogenesis does not initiate malignancy itself but can promote tumour progression and metastasis; therefore, many therapeutic strategies have been investigated to inhibit angiogenesis in cancer over the past decades (Ferrara et al. 2004). Vascular endothelial growth factor receptor 2 (VEGFR2), also known as kinase insert domain receptor (KDR), is a major mediator of VEGF’s biological effects, and plays a vital role in tumour angiogenesis (Olsson et al. 2006). Targeting VEGF/VEGFR2 and the downstream signal molecules, such as AKT/PKB and MAPK family, can inhibit angiogenesis and hinder tumour growth (Ferrara 2005).

Berberine, a benzylisoquinoline alkaloid mainly isolated from *Rhizoma coptidis* [*Coptis chinensis* Franch. (Ranunculaceae)], has been used in traditional Chinese medicine for thousands of years to treat diarrhoea and diabetes (Sun et al. 2009). More and more studies in recent years have shown that berberine also exerts inhibitory activity on many types of cancer, such as glioblastoma, colorectal, lung, prostate and ovarian (Peng et al. 2006; Eom et al. 2008; Meeran et al. 2008; Jin et al. 2015; Liu et al. 2015). In previous studies reported with human glioblastoma cells, berberine induced autophagy by targeting the AMPK/mTOR/ULK1 pathway, induced senescence by downregulating the EGFR/MEK/ERK pathway, and induced G1 arrest and apoptosis through mitochondrial/caspases pathway (Eom et al. 2008; Liu Q et al. 2015; Wang et al. 2016). However, an antiangiogenic mechanism has not been demonstrated. In this study, we tested inhibitory activity of berberine on angiogenesis in both cell-based assays and a mouse xenograft model of human glioblastoma, as well as clarified involvement of the VEGFR2/ERK pathway.

## Materials and methods

### Reagents

Berberine was purchased from Sigma-Aldrich (St. Louis, MO). Berberine powder was dissolved in phosphate buffered saline (PBS), then sterilized using a 0.22 μm pore filter (Millipore, Billerica, MA) and stored at 4 °C until use.

### Cell culture

U87 and U251 human glioblastoma cell lines were obtained from American Type Culture Collection (Manassas, VA), and maintained in RPMI1640 medium supplemented with 10% foetal bovine serum (FBS), 100 U/mL penicillin and 100 μg/mL streptomycin (Gibco, Grand Island, NY). Primary human umbilical vein endothelial cells (HUVEC) were purchased from ScienCell (Carlsbad, CA) and maintained in MCDB131 medium (PAA, Pasching, Austria) with 1% endothelial cell growth supplement (ScienCell), 5% FBS, 100 U/mL penicillin and 100 μg/mL streptomycin. All cells were cultured in a humidified 5% CO_2_ air atmosphere at 37 °C. All experiments were performed using cells within eight passages after receipt.

### Cell viability

U87 or U251 cells were trypsinized and plated in 96-well cell culture plates at the concentration of 5 × 10^3^ cells per well. Twenty-four h later, the medium was removed and replaced with fresh medium with or without berberine (6.25, 12.5, 25, 50, 100 and 200 μmol/L). After 48 h, cell viability was determined by using 3-(4,5-dimethylthiazol-2-yl)-2,5-diphenyltetrazolium bromide (MTT, Amresco, Solon, OH) following the manufacturer’s instructions. The absorbance of converted dye was measured at the wavelength of 490 nm and the absorbance was directly proportional to cell viability. The reference wavelength was 630 nm.

### Cell proliferation

U87 or U251 cells were trypsinized and plated in 96-well cell culture plates at the concentration of 5 × 10^3^ cells per well. The medium was removed 24 h later, and replaced with fresh medium with or without berberine (25, 50 and 100 μmol/L). After 48 h, cell proliferation was determined by using EdU assay kit (RiboBio, Guangzhou, China) following the manufacturer’s instructions. The nuclei of proliferated cells were dyed red with the kit, while the nuclei of all cells were dyed blue with 4′,6-diamidino-2-phenylindole (DAPI). The number of dyed nuclei was counted using the software ImageJ (NIH, Bethesda, MD).

### Cell migration

HUVEC were seeded into the insert of Transwell (Corning, Tewksbury, MA) at the concentration of 1 × 10^5^ cells per well, and then cultured in serum-free culture media. Berberine (50 μmol/L) or control (PBS) was added to the lower reservoirs. Cells were subsequently allowed to migrate across polycarbonate filter for 12 h at 37 °C. Non-migrated cells on the top side of the filter were removed by scraping. Migrated cells on the bottom side of the filter were subsequently fixed with 4% paraformaldehyde for 30 min and stained by haematoxylin solution (Beyotime, Shanghai, China) for 5 min. The number of stained cells in five random fields of each well was counted using the software ImageJ to determine the average number of migrated cells.

### Tube formation

Twenty-four-well plates were coated with 300 μL Matrigel (BD, San Jose, CA) per well and incubated at 37 °C for 20 min to allow the Matrigel to solidify. HUVEC were plated at the concentration of 1 × 10^5^ cells per well and incubated with berberine (50 μmol/L) or PBS at 37 °C for 6 h. The cells were then photographed using a Zeiss digital camera. Five randomly selected fields per well were photographed. Tube formation was quantified by measuring the length of capillary structures using the software ImageJ. The average value of tube length in five fields was taken as the value for each sample.

### Animals

Athymic nude mice (5- to 6-weeks-old) were obtained from Charles River Laboratories (Beijing, China). Animals were housed in a temperature-controlled room (22 °C) with 12 h light/dark cycling under pathogen-free conditions, and had free access to food and water. All experimental procedures related to the animals complied with the ‘Guide for the Care and Use of Laboratory Animals’ published by the National Institutes of Health of the United States and were approved by Institutional Animal Care and Use Committee of Zhujiang Hospital. All mice were randomly divided into two groups. One of the groups was treated with berberine (50 mg/kg) by oral gavage, the other was treated with vehicle (carboxymethylcellulose sodium). The treatment was started seven days prior to cell implantation and lasted until the end of the experiment.

### Ectopic xenograft model

U87 cells were harvested by trypsin/EDTA treatment and washed with cold PBS before centrifugation, then the cells were re-suspended in PBS and kept on ice before used. Tumour cells (1 × 10^6^ cells in 0.1 mL PBS) were injected subcutaneously into the right flank of the mice. Tumour diameter was measured every four days by caliper, and tumour volume was calculated by the formula: 0.5×(larger diameter)×(smaller diameter)^2^. At the end of the experiment, the animals were sacrificed by CO_2_ euthanasia and their tumour tissues were harvested, weighted, and then stored in –80 °C for further analysis.

### Orthotopic xenograft model

U87 cells were harvested by trypsin/EDTA treatment and washed with cold PBS before centrifugation. Tumour cells (2 × 10^5^ cells in 5 μL of methylcellulose) were injected intracranially into the mice by stereotactic surgery (2 mm left and 1 mm anterior to the bregma, 2 mm deep from the dura). The experiment was terminated when the mice in the vehicle group become moribund, and all death dates were recorded. At the end of the experiment, the animals were sacrificed by CO_2_ euthanasia.

### Haemoglobin assay

Concentration of haemoglobin in tumour tissue was determined using Drabkin’s Reagent (Sigma, St. Louis, MO) by a colorimetric method according to the manufacturer’s instructions.

### Real-time PCR

Trizol reagent (Takara, Dalian, China) was used for isolating total RNA of tumour tissue. Tissue (50–100 mg) was directly lysed by mixing with 1 mL of Trizol reagent and homogenized using a homogenizer. Then, 0.2 mL of chloroform was added to the homogenized sample, and incubated for 15 min at room temperature. Subsequently, RNA was precipitated by mixing with isopropyl alcohol. Total RNA yield was quantified by UV spectrophotometry measured at 260 nm. Then mRNA was isolated from total RNA by using Oligo (dT), and reverse transcribed into first-strand complement DNA (cDNA) and amplified using a PrimeScript 1st strand cDNA synthesis kit (Takara, Dalian, China). Reaction system included 2 μL of cDNA, 12.5 μL of 2 × SYBR Green 1 Master Mix (Takara, Dalian, China), and 1 μL of each primer. The PCR condition was as follows: pre-incubation at 95 °C for 30 s, followed by 40 cycles of denaturation at 95 °C for 5 s, and annealing/extension at 60 °C for 30 s using iQ5 Real-Time PCR detection system (Bio-Rad, Hercules, CA). The primers are listed in Table 1.

**Table 1.** Primers for real-time PCR.

CD31	5′-TATCCAAGGTCAGCAGCATCGTGG-3′
	5′-GGGTTGTCTTTGAATACCGCAG-3′
18S	5′-GATGGGCGGCGGAAAATAG-3′
	5′-GCGTGGATTCTGCATAATGGT-3′

### Western blot

Tumour tissue was rinsed in ice-cold PBS, and lysed in lysis buffer (Beyotime, Shanghai, China). Equal amounts of protein were separated by 10% SDS-PAGE, transferred to PVDF membrane (Millipore, Billerica, MA), and blocked with 5% nonfat dry milk in TBS-Tween 20 for 1 h at room temperature. The membrane was incubated with primary antibody overnight. Antibodies to p-VEGFR2, VEGFR2, p-ERK, ERK, p-p38, p38, p-JNK, JNK and GAPDH were purchased from Cell Signaling Technology (Danvers, MA). After washing, the membrane was incubated with horseradish peroxidase-conjugated secondary antibody (Cell Signaling Technology, Danvers, MA) for 1 h. Following several washes, the blots were developed by Immobilon solution (Millipore, Billerica, MA).

### Statistical analysis

Data were analysed and graphed by Prism 6.0 (GraphPad Software, La Jolla, CA), and presented as mean ± standard deviation (SD). Significance of difference between groups was analysed by performing two-way RM ANOVA for time course study, or one-way ANOVA with Dunnett’s multiple comparison test or unpaired Student's *t*-test for other studies. Survival studies were assessed using the Kaplan–Meier survival curves and analysed with the Mantel–Cox log-rank test. *p* Value< 0.05 was considered statistically significant.

## Results

### Berberine inhibited cell viability and proliferation of human glioblastoma cell lines

**Figure 1.**
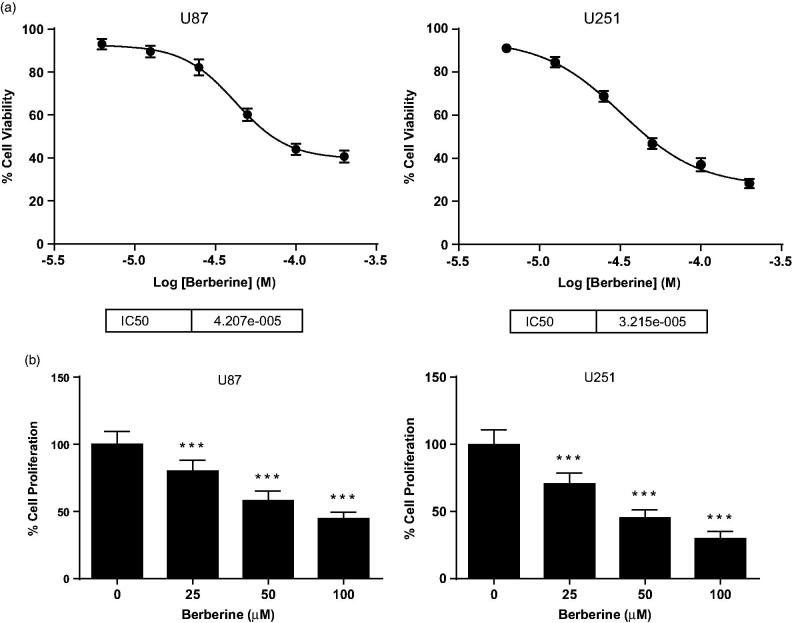
Cell viability and proliferation of human glioblastoma cells. (A) U87 (left) and U251 (right) cells were treated with 6.25, 12.5, 25, 50, 100 and 200 μmol/L of berberine for 48 h before cell viability was determined by MTT assay. (B) U87 (left) and U251 (right) cells were treated with 25, 50 and 100 μmol/L of berberine for 48 h before cell proliferation was determined by EdU assay. ***p< 0.001 vs. control. All experiments were repeated at least three times.

We treated U87 and U251 human glioblastoma cells with 6.25, 12.5, 25, 50, 100 and 200 μmol/L of berberine, and found that berberine could dose dependently inhibit cell viability of both U87 and U251 cells (Figure 1(A)). The IC_50_ values for U87 and U251 were 42 and 32 μmol/L, respectively. Then, we performed EdU assay to determine antiproliferation effect of berberine on glioblastoma cells. Compared with PBS control, 50 μmol/L of berberine could significantly inhibit proliferation of U87 and U251 cells, with inhibition rates of 42.00 ± 7.07% and 54.83 ± 6.18%, respectively (Figure 1(B)).

### Berberine inhibited cell migration and tube formation of HUVEC

**Figure 2.**
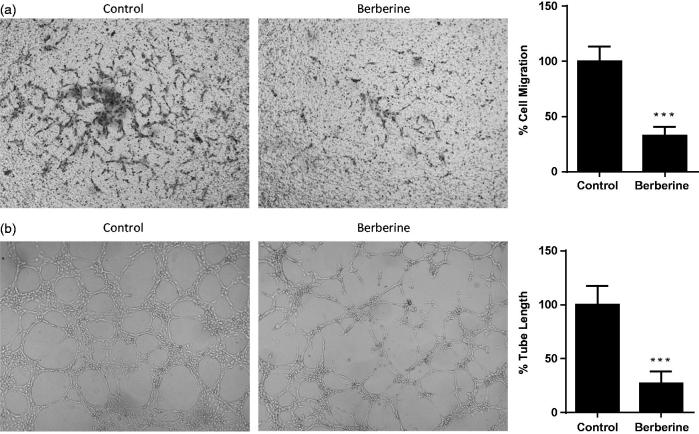
Cell migration and tube formation of HUVEC. (A) HUVEC was seeded into Transwell and treated with 50 μmol/L of berberine for 12 h. (B) HUVEC was seeded into Matrigel and treated with 50 μmol/L of berberine for 6 h. ***p< 0.001 vs. control. All experiments were repeated at least three times.

Next, we determined inhibitory effects of berberine on *in vitro* angiogenesis of endothelial cells. In migration assay, 50 μmol/L of berberine reduced the number of HUVEC which migrated to the other side of Transwell filter by 67.50 ± 8.14% (Figure 2(A)). In tube formation assay, 50 μmol/L of berberine significantly inhibited tube formation of HUVEC in Matrigel, a concentrated extracellular matrix, in which the tube length was reduced by 73.00 ± 11.12% (Figure 2(B)).

### Berberine inhibited angiogenesis in glioblastoma xenografts

**Figure 3.**
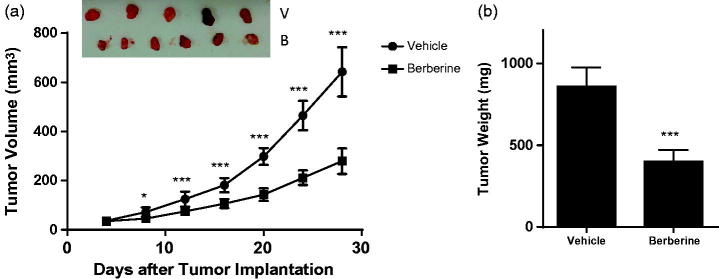
Tumour growth in ectopic xenograft model of glioblastoma. Athymic nude mice were injected subcutaneously with U87 cells (1 × 10^6^ cells in 0.1 mL PBS), and treated with vehicle (carboxymethylcellulose sodium) or 50 mg/kg of berberine by oral gavage for 28 days. (A) Tumour volume. (B) Tumour weight. ***p< 0.001 vs. vehicle group. *p< 0.05 vs. vehicle group. *N* = 6 for each group.

**Figure 4.**
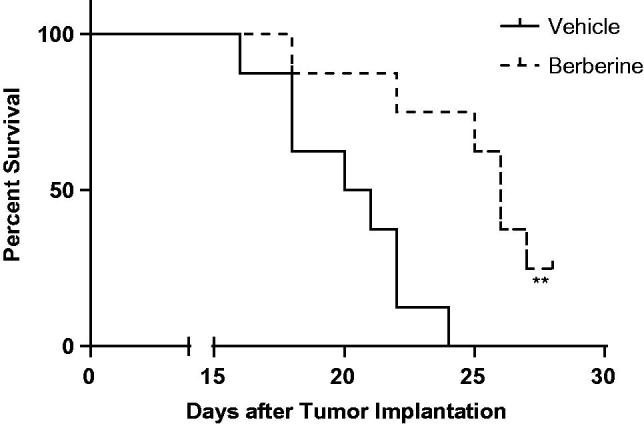
Tumour growth in orthotopic xenograft model of glioblastoma. Athymic nude mice were injected intracranially with U87 cells (2 × 10^5^ cells in 5 μL of methylcellulose) by stereotactic surgery, and treated with vehicle (carboxymethylcellulose sodium) or 50 mg/kg of berberine by oral gavage for 28 days. Survival studies were assessed using the Kaplan–Meier survival curves and analysed with the Mantel–Cox log-rank test. *N* = 8 for each group.

In ectopic model, 50 mg/kg of berberine could significantly decrease the tumour volume from day 8 post-implantation (Figure 3(A)). At the termination, tumour weight in berberine group was 401.2 ± 71.5 mg, while that in vehicle group was 860.7 ± 117.1 mg (*p* ˂ 0.001, Figure 3(B)). In orthotopic model, berberine treatment could significantly improve the survival rate of mice (*p*= 0.0078, Figure 4).

**Figure 5.**
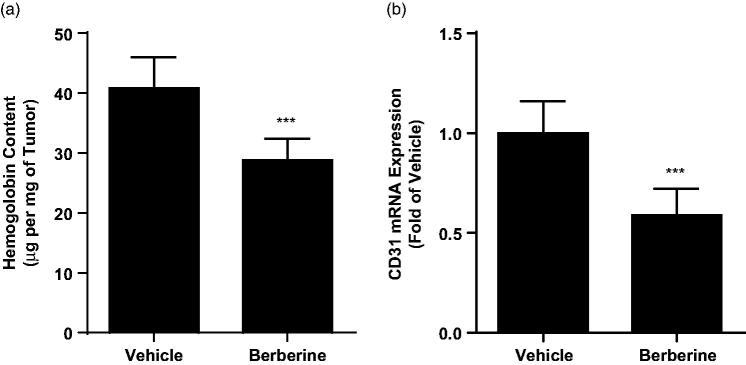
Angiogenesis analysis in tumour tissue from ectopic xenograft model. (A) Haemoglobin content determined by colorimetric method. (B) mRNA expression of CD31 determined by real-time PCR. ***p< 0.001 vs. vehicle group. *N* = 6 for each group.

Next, we analysed the vascular density in tumour tissue from ectopic xenograft by haemoglobin assay and found that berberine treatment could significantly decreased haemoglobin content from 40.84 ± 5.15 to 28.81 ± 3.64 μg/mg (*p* ˂ 0.001, Figure 5(A)). Likewise, we also observed declined mRNA expression level of CD31, biomarkers of endothelium, in tumour tissue after berberine treatment (*p* ˂ 0.001, Figure 5(B)). These data showed that berberine inhibited angiogenesis in glioblastoma xenografts.

### Berberine inhibited angiogenesis by targeting the VEGFR2/ERK pathway

**Figure 6.**
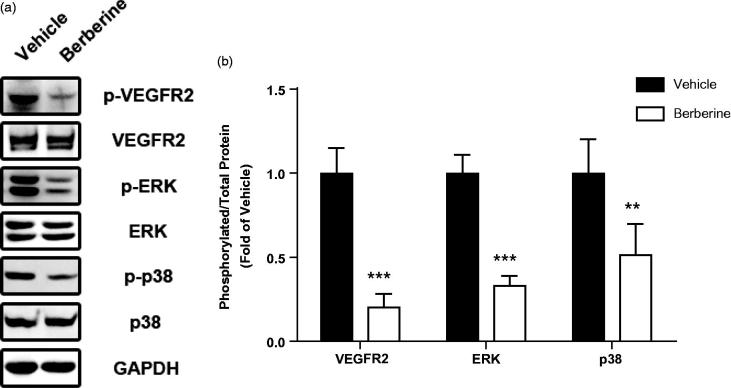
Molecular mechanisms involved in antiangiogenic effect of berberine. Tumour tissue from ectopic xenograft model was isolated and homogenized for Western blot analysis. ***p< 0.001 vs. vehicle group, **p< 0.01 vs. vehicle group. *N* = 6 for each group.

In order to investigate possible molecular mechanisms in berberine-induced angiogenesis inhibition, we analysed protein expression of VEGFR2 and MAPK pathways by Western blots. Total expression of VEGFR2 was not changed after berberine treatment, while phosphorylation of VEGFR2 was significantly reduced (*p* ˂ 0.001, Figure 6). Likewise, phosphorylation of ERK and p38 was also reduced after berberine treatment (*p* ˂ 0.001 and *p* ˂ 0.01, respectively, Figure 6). However, total expression and phosphorylation of JNK, another member of MAPK family, was not changed by berberine administration (data not shown).

## Discussion

Over the past decades, the incidence of glioblastoma has rapidly increased. In spite of advances in neurosurgical techniques and radiotherapy, glioblastoma has retained its dismal prognoses (Omuro and Deangelis 2013). Surgery and radiotherapy are limited by the infiltration of tumour cells into healthy brain, leading to recrudesce even after treatment. Adjuvant chemotherapy is thus essential for the treatment of glioblastoma (Preusser et al. 2011). Compared with conventional chemotherapy, berberine has much less side effects on the patients. In a meta-analysis of the safety of berberine on 27 randomized controlled clinical trials which included 2569 patients, no serious adverse effect was reported (Lan et al. 2015). In our study, we reported translational research of berberine on glioblastoma xenograft, shedding new light on complementary and alternative therapy for this disease.

Targeting angiogenesis to prevent tumour progression is a new direction for cancer therapy (Ferrara et al. 2004). Tumour angiogenesis involves several sequential phases, including proliferation and secretion of tumour cells as well as cell migration and tube formation of endothelial cells (Ausprunk and Folkman 1977; Bao et al. 2006). In previous study, Liu reported that berberine inhibited viability of U87 cells with IC_50_ of 21.76 μmol/L after 72 h treatment (Liu et al. 2015). Here, we observed an IC_50_ of 42 μmol/L using the same cell line but with 48 h treatment. Wang et al. (2016) also reported a similar IC_50_ with the same condition as ours. Besides, in these two studies, berberine was also found to inhibit proliferation of U87 and U251 cells, which was confirmed by our study. Lin et al. (2004) and Jie et al. (2011) reported inhibitory effects of berberine on cell migration in Transwell and tube formation in Matrigel; Hamsa and Kuttan (2012) reported inhibitory effects of berberine on cell migration in scrape-wound. These results were consistent with our *in vitro* data. More important, we first disclosed antiangiogenic effect of berberine in glioblastoma xenografts, in which haemoglobin assay and CD31 analysis directly proved that berberine reduced vascular density in the tumour.

In two independent studies on patient tumour samples using fluorescence *in situ* hybridization, amplification of gene encoding VEGFR2 receptor tyrosine kinase was frequent in glioblastoma (Joensuu et al. 2005; Puputti et al. 2006). Besides, interference with VEGF-VEGFR2 signalling potentiated the ionizing radiation-induced tumour cell death, supporting the candidacy of this signalling cascade as a therapeutic target, possibly in combination with radiotherapy (Knizetova et al. 2008). Ma et al. (2017) reported that berberine downregulated the phosphorylation of VEGFR2 in ZR-75-30 breast cancer cells, while Jie et al. (2011) reported that berberine downregulated VEGF mRNA expression and prevented secretion of VEGF from Hep G2 cells. In our study, berberine significantly reduced the phosphorylation of VEGFR2 in tumour tissue from glioblastoma xenograft, providing *in vivo* evidence for VEGFR2-targeted glioblastoma therapy.

MAPK family, including ERK, p38 and JNK, are critical signal molecules downstream of VEGFR2 to transmit signal to promote cell survival, migration and angiogenesis (Olsson et al. 2006). Inhibition of the ERK pathway during the migratory phase of endothelial cells resulted in loss of bipolarity, detachment, survival of the cells and retraction of sprouting tubules (Mavria et al. 2006). Some synthetic molecules were designed to target ERK pathway to inhibit tumour angiogenesis and treat different types of cancer (Wilhelm et al. 2004). Berberine was reported to reduce phosphorylation of ERK in HUVEC, U87 glioblastoma cells, ZR-75-30 breast cancer cells and SCC-4 tongue squamous cancer cells (Gao et al. 2009; Ho et al. 2009; Liu et al. 2015; Ma et al. 2017). Here, we observed similar reduction of ERK phosphorylation in tumour tissue from glioblastoma xenograft. Furthermore, p38 phosphorylation was also reduced while neither phosphorylation nor total JNK was changed in our study. All data suggested VEGFR2/ERK pathway was involved in antiangiogenic effect of berberine.

## Conclusions

We reported translational research of berberine in glioblastoma therapy, and proved inhibitory activity of berberine on angiogenesis in both cell-based assays and mouse xenograft model of human glioblastoma, as well as clarified involved VEGFR2/ERK pathway. All these data shed new light on complementary and alternative therapy for this disease.
